# Eco-epidemiological study of an endemic Chagas disease region in northern Colombia reveals the importance of *Triatoma maculata* (Hemiptera: Reduviidae), dogs and *Didelphis marsupialis* in *Trypanosoma cruzi* maintenance

**DOI:** 10.1186/s13071-015-1100-2

**Published:** 2015-09-22

**Authors:** Omar Cantillo-Barraza, Edilson Garcés, Andrés Gómez-Palacio, Luis A. Cortés, André Pereira, Paula L. Marcet, Ana M. Jansen, Omar Triana-Chávez

**Affiliations:** Grupo BCEI, Universidad de Antioquia UdeA, Calle 70 No. 52-21, Medellin, Colombia; Unidad de Entomología Médica, Secretaría de Salud del Departamento de Bolívar, Cartagena, Colombia; Laboratory of Trypanosomatid Biology, Oswaldo Cruz Institute, FIOCRUZ, Av. Brazil 4365, CEP 21040-360 Rio de Janeiro, RJ Brazil; Centers for Disease Control and Prevention (CDC), Division of Parasitic Diseases and Malaria, Entomology Branch, Atlanta, GA USA

**Keywords:** Colombia, Risk factors for Chagas disease, *Trypanosoma cruzi*, Seroprevalence, Dogs, Non-domiciliated vectors

## Abstract

**Background:**

In Colombia, *Rhodnius prolixus* and *Triatoma dimidiata* are the main domestic triatomine species known to transmit *T. cruzi*. However, there are multiple reports of *T. cruzi* transmission involving secondary vectors. In this work, we carried out an eco-epidemiological study on Margarita Island, located in the Caribbean region of Colombia, where Chagas disease is associated with non-domiciliated vectors.

**Methods:**

To understand the transmission dynamics of *Trypanosoma cruzi* in this area, we designed a comprehensive, multi-faceted study including the following: (i) entomological evaluation through a community-based insect-surveillance campaign, blood meal source determination and *T. cruzi* infection rate estimation in triatomine insects; (ii) serological determination of *T. cruzi* prevalence in children under 15 years old, as well as in domestic dogs and synanthropic mammals; (iii) evaluation of *T. cruzi* transmission capacity in dogs and *Didelphis marsupialis,* and (iv) genetic characterization of *T. cruzi* isolates targeting spliced-leader intergene region (SL-IR) genotypes*.*

**Results:**

Out of the 124 triatomines collected, 94 % were *Triatoma maculata*, and 71.6 % of them were infected with *T. cruzi.* Blood-meal source analysis showed that *T. maculata* feeds on multiple hosts, including humans and domestic dogs. Serological analysis indicated 2 of 803 children were infected, representing a prevalence of 0.25 %. The prevalence in domestic dogs was 71.6 % (171/224). Domestic dogs might not be competent reservoir hosts, as inferred from negative *T. cruzi* xenodiagnosis and haemoculture tests. However, 61.5 % (8/13) of *D. marsupialis*, the most abundant synanthropic mammal captured, were *T. cruzi-*positive on xenodiagnosis and haemocultures.

**Conclusions:**

This study reveals the role of peridomestic *T. maculata* and dogs in *T. cruzi* persistence in this region and presents evidence that *D. marsupialis* are a reservoir mediating peridomestic-zoonotic cycles. This picture reflects the complexity of the transmission dynamics of *T. cruzi* in an endemic area with non-domiciliated vectors where active human infection exists. There is an ongoing need to control peridomestic *T. maculata* populations and to implement continuous reservoir surveillance strategies with community participation.

**Electronic supplementary material:**

The online version of this article (doi:10.1186/s13071-015-1100-2) contains supplementary material, which is available to authorized users.

## Background

Chagas disease is a zoonosis endemic to the Americas. *Trypanosoma cruzi,* its aetiological agent, is a multi-host parasite adaptable to hundreds of mammalian species in diverse ecological niches that uses numerous routes of transmission [1]. *T. cruzi* is defined as a heterogeneous taxon and subdivided into six Discrete Typing Units (DTUs), named TcI to TcVI [2], and more recently a new genotype related with bats designed as TCbat [3]. Moreover, within TcI large intraspecific genetic diversity has been recently reported, and the existence of genotypes associated with domestic (TcIa), peridomestic (TIb) and sylvatic (TcId) transmission cycles was suggested [4]. The incorporation of *T. cruzi* to domestic transmission cycles is carried out by the presence of triatomine vector species adapted to human dwellings and by the random house invasion of sylvatic adult triatomines, mechanisms by which persistent Chagas transmission occurs along the American continent [4].

In Colombia, *Rhodnius prolixus* and *Triatoma dimidiata* are the main domestic species known to transmit *T. cruzi* [5]. However, there are multiple reports of *T. cruzi* transmission involving secondary or non-domestic adventitious household-invading triatomines, such as *R. pallescens, Eratyrus cuspidatus*, *T. venosa* and *T. maculata;* which suggest the importance of the sylvatic cycle and a close relationship between the zoonotic cycles of *T. cruzi* and human populations [5–10].

The epidemiological importance of some secondary vectors in Colombia has been supported by showing their involvement in *T. cruzi* transmission in conventional vectorial or oral transmission [11–13]. Among them, *T. maculata,* was often considered to be of little epidemiological relevance due to its low infection rates and apparently ornithophilic preferences [5, 7, 9]. However, recent studies in the Colombian Caribbean region reported the occurrence of active transmission, and infection risk was associated with highly infected *T. maculata* in peridomestic human dwellings [6, 10, 14, 15]. The differential relevance of *T. maculata* in transmission cycles from distinct geographical areas, suggests the ability of this vector to adapt to a variety of environments and its distinct ecological behaviours have different epidemiological implications [7, 16]. The eco-epidemiological importance of *T. maculata* might vary according to its ability to feed on different mammals and by the local fauna composition, the rate of host-vector contact, and the transmissibility competence of domestic, synanthropic and wild mammals that serve as blood meal sources for this species [1, 16–19].

Several studies have highlighted the importance of peridomestic habitats in the re-infestation process by domestic and secondary vectors, oral outbreaks and emergence or re-emergence of Chagas disease in a region [16, 17, 19, 20]. The evaluation of infection levels and the eco-epidemiological roles of domestic, synanthropic and intrusive sylvatic mammals is essential for understanding the epidemiological profile of the disease, the intensity of *T. cruzi* transmission near homes and the local risk for emergence of human cases [1]. The serological evaluation of domestic mammals is the easiest way to estimate the proximity of *T. cruzi* transmission cycles to humans. Dogs-as-sentinels programmes have been proposed for evaluating transmission risk and exposure to *T. cruzi* as it may indicate *T. cruzi* transmission hotspots [1, 17, 21].

In Colombia, it has been estimated that 0,96 % of the population living in endemic areas is infected, representing approximately 437,960 people [22], although this value is variable across a country that includes complex eco-epidemiological local scenarios. Furthermore, the role of domestic dogs as elements in the eco-epidemiology of Chagas disease transmission has been determined in places where the transmission to humans involves domestic vectors like *R. prolixus,* such as the regions of Boyacá and Tolima [23–26]. However, little work has been done to characterize the *T. cruzi* transmission dynamics in areas where only non-domiciliated triatomines are present. Aiming to understand this, we performed a multi-faceted eco-epidemiological study involving the following: vector identification; blood-meal source determination; natural infection rate estimation; seroprevalence estimation in children, dogs and synanthropic mammals; and *T. cruzi* genotyping. Our results demonstrate the role of *T. maculata* as vector, dogs as bioindicators of *T. cruzi* persistence in peridomestic ecotopes, and *D. marsupialis* as a reservoir connecting the zoonotic/peridomestics ecotopes. These factors constitute the principal eco-epidemiological contributors to the risk of human infection in this region.

## Methods

### Study area and sampling

The study was carried out between 2010 and 2012 in seven locations from two municipalities in lowland Margarita Island in the Bolivar department of the Caribbean plains bioregion of Colombia. Sampled localities were Tierrafirme (TF), La Rinconada (LR), La Loma de Simon (LS) and Guataca (GU) located in rural zones of the Mompós municipality; and Talaigua Nuevo (TN), Talaigua Viejo (TV) and El Vesubio (EV) located in the Talaigua Nuevo municipality (geographical location in Additional file 1). The study area is a tropical sub-humid region surrounded by cattle pastures, crops and patches of native forest. The population is approximately 30,000 inhabitants, and the economy depends on subsistence agriculture. Previous studies performed in Talaigua Nuevo and Mompós, reported the presence of infected household-invading *T. maculata* and *R. pallescens* [6, 15].

## Insect collection

### Households

Triatomine insect searches were carried out in collaboration with local personnel from the Ministry of Health. Domiciliary and peridomiciliary collections were carried out with the traditional manual collection method using a dislodging spray [27]. A total of 483 houses were inspected in the 7 localities. All specimens were identified to species following the morphological keys of Lent and Wygodzinsky [28], and stored at 4 °C until further processing.

### Surveillance program

Community participation was defined as the involvement of local residents in collecting insects in their household and neighbourhoods. Community-based surveillance was performed by academic personnel (high school students and teachers) from Mompós municipality, the Association of Community Mothers of Talaigua Nuevo (ACMTN), leaders of Community Action Committees (CAC) and the Families in Action Program (FAP), summoned by Talaigua Nuevo town hall. Workshops and individual visits to houses were organized to provide information about Chagas disease and vectors. Local residents were then instructed to collect triatomines found inside their houses and neighbourhoods in plastic vials/bags labelled with their name, address and date of capture. All participants received biosafety training and gloves for self-protection when manipulating the insects.

Receiving stations for deposit and storage of triatomine insects were established in schools at all localities and in the office of the health secretary in the TN city hall. Insects were gathered from the school or health secretary each month. Additionally, the houses that delivered nymphs were visited by technical staff to verify the presence of insect colonies. Collected insects were sent to a laboratory in Medellin for species identification, *T. cruzi* infection determination and blood-meal source analysis.

### *T. cruzi* triatomine infection

All triatomines received alive were inspected for *T. cruzi* infection by direct microscopy and molecular methods. Faeces were obtained by abdominal compression and diluted in 300 μL of sterile PBS pH 7.2. A 10-μL aliquot was examined under an optical microscope at 400X for flagellated forms. The remaining sample was used for DNA extraction and *T. cruzi* genetic typing (see below).

### Blood-meal source determination

To identify blood-meal sources of *T. maculata*, 40 adults were selected according to their apparent nutritional status (i.e., insects that appeared to have recently fed). We carried out a cytochrome b gene high-resolution melting (HRM) analysis from insect faeces following standard procedures reported elsewhere [18, 29]. Briefly, the cyt b gene was amplified using the primers forward (5′-CCCCTCAGAATGATATTTGTCCTCA- 3′) and reverse (5′-CCATCCAACATCTCAGCATGATGAAA-3′) [30]. Reaction conditions and thermal profiles were performed as described previously [18]. The standard host species used in this study were opossum (*D. marsupialis*), mouse (*Mus musculus* L), cow (*Bos taurus*), goat (*Capra aegagrus*), dog (*Canis lupus familiaris*) sheep (*Ovis aries*), horse (*Equus caballus*), pig (*Sus scofa*), rabbit (*Oryctolagus cuniculus*), cat (*Felis catus*), rat (*Rattus norvegicus*), donkey (*Equus asinus*), human (*Homo sapiens* L) and chicken (*Gallus* L). These species were selected by DNA availability, proximity to human environments, and ecoepidemiological importance.

### Survey and analytical approaches

A questionnaire was provided to dog owners to assess epidemiological data and risk factors associated with Chagas disease [27]. For each pet, the following information was collected: name, sex, age, the pet’s main function, general physical condition, birth place, history of visiting or residing outside the study area, reproductive data and behavioural details. Household variables surveyed by direct observation included the presence or absence of shacks or exterior storage buildings for rearing domestic birds and the presence of coconut or *A. butyracea* palm trees. Data management and statistical analyses were conducted using SPSS V.15.0 and Epi-info V 2000. A screening (bivariate analysis) was performed using 2 x 2 contingency tables, using dogs infected and exposure variables as dependent variables.

### Mammal hosts: domestic and synanthropic sample collection

A total of 244 dogs (out of an estimated ~2000 in the study area) were analysed, assuming an estimated *T. cruzi* prevalence of ~15 % and a margin of error of ~5 % (Table 1). Inclusion criteria for selected dogs were as follows: (i) born and raised in the study area, (ii) having a recognizable owner, and (iii) information available about the animal’s history (i.e., age, rest sites, feeding frequency and general health). With the informed consent of their owners, blood samples were drawn from the dorsal-tibial or radial veins under minimal stress and with the help of the owner. The samples were centrifuged and serum was stored at −20 °C for serological assays.

**Table 1:** Summary of capture and infection in triatomine insects by *T. cruzi,* and seroprevalecence in dogs

			Peridomiciliary hosts						Vectors			
			Dogs		Synanthropic mammals				Triatomine insects		Human screening	
					Didelphis		Rattus					
Municipality	Locality	Evaluated Houses	n	IR	n	Infection rate	n	Infection rate	*T. maculata*	Other species	n	% infection
Mompós	Tierrafirme (TF)	80	39	71.7	6	50	0	ND	14	1	112	0.89
	La Rinconada (LR)	90	38	68.4	3	33	0	ND	8	2	81	0
	La Loma de Simon (LS)	50	8	62.5	2	100	0	ND	8	0	100	1
	Guataca (G)	60	ND	ND	ND	ND	0	ND	2	0	70	0
Talaigua	Talaigua Nuevo (TN)	140	86	69.7	2	100^a^	10	0	81	1	290	0
	Talaigua Viejo (TV)	40	36	77.7	0	0	0	ND	2	2	69	0
	El Vesubio (V)	50	37	64.8	0	0	0	ND	2	1	81	0
Total		510	244	71.3	13	61,54	10	0	117	7	803	0.25

*T. cruzi i*nfection in small mammalian fauna and humans was examined in the study areas Tierrafirme (TF), La Rinconada (LR), La Loma de Simon (LS), Guataca (G), Talaigua Nuevo (TN), Talaigua Viejo (TV), El Vesubio (EV)

^a^One of them with positive infection in a scent gland

Small wild and synanthropic mammals were captured using Tomahawk® and Sherman® live traps arranged linearly. The capture points were established with intercalated traps. The traps were baited with a mixture of peanut, banana, oat and fish, and sets of 15 live traps were placed at 20 m intervals. The traps were placed in peridomestic areas that we defined as the total area around the house, including permanent or temporary structures built and used by humans or their domestic animals. The total capture effort was 360 trap-nights for each town. Eight collection periods were conducted over two consecutive years (2010 and 2011). Wild mammals were anesthetized intramuscularly (9:1 ketamine clorohidrate 10 % and xylazine 2 %) and had their blood collected by cardiac puncture. All of the tested mammals were returned to the wild at the capture site after the experimental procedure.

### Serological diagnosis in dogs

Detection of anti-*T. cruzi* antibodies was conducted using an Indirect Immunofluorescence Antibody Test (IFAT) and an Enzyme-Linked Immunosorbent Assay (ELISA, Bio-Manguinhos, FIOCRUZ, Rio de Janeiro, Brazil). The test cut-off was determined as of 1/40 for IFAT and optical absorbance ≥0.200 (mean +/− 3SD) for the ELISA test. Animals were defined as seropositive when samples were reactive to both tests.

Cross-reactions and/or mixed infection by *T. cruzi* and *Leishmania* spp. in serum were also assayed with antigens derived from a mixture of *L. infantum* and *L. panamensis* using IFAT and the Rapid Test for Diagnosis of Canine Visceral Leishmaniasis (TR DPP®, Bio-Manguinhos, FIOCRUZ, Rio de Janeiro, RJ, Brazil-DPP) [31, 32].

The IFAT cut-off value adopted for *T. cruzi* infection was 1/40 when the IFAT results for *L. infantum* was lower than 1/40 and the DPP result was negative. On the other hand, in dogs positive for *L. infantum*, positive *T. cruzi* infection was considered only when the IFAT titer was 1/80 or higher. For *L. infantum* infection, the adopted IFAT cut-off value was 1/40 when the infection was also confirmed by DPP and 1/80 when the DPP assay was negative [31, 32].

### *T. cruzi* detection in domestic dogs and synanthropic mammals

Small mammals were anesthetized (9:1 ketamine cloridrate 10 % and xylazine 2 %) to obtain blood samples, which were examined using xenodiagnosis and haemoculture. Similarly, a group of 20 *T. cruzi* sero*-*positive dogs showing high antibody titers were selected because higher IgG titers occur in the initial phases of infection [33]. These dogs were re-examined using xenodiagnosis and haemoculture three months after blood collection. For each animal, two tubes containing NNN medium covered with an overlay of LIT were inoculated with between 0.3 and 0.6 mL of blood. Tubes were examined weekly for the presence of *T. cruzi* epimastigote forms for three months. Xenodiagnostic tests were performed using 20 fourth or fifth instar *R. prolixus* nymphs per dog and 5–10 nymphs for small mammals. *R. prolixus* faeces were subsequently examined four times in the next 60 days by direct observation at 400X.

Additionally, secretions from the scent gland of a captured *Didelphis marsupialis* was collected by direct manipulation and observed under the MO in search of moving flagellates. The positive sample was then stored at 4 °C, for *T. cruzi* detection by molecular markers and posterior genetic typing (see below).

The animal protocol was reviewed and approved by the Institutional Animal Care and Use Committee of the University of Antioquia, Colombia (License 08-012-185, 42-12/05/2008).

### DNA extraction and *T. cruzi* genetic typing

Total DNA was extracted from faeces of *T. maculata* and *R. pallescens* captured in domiciliary and peridomiciliary habitats and from the *R. prolixus* used in xenodiagnosis using a phenol-chloroform method [34]. DNA was also extracted from the glandular secretions of the *D. marsupialis* that was positive in MO.

*T. cruzi* molecular detection was performed by direct PCR of a 180 bp fragment of the satellite DNA using the primers TcZ1 and TcZ2 [35] and a 330-bp DNA fragment of the variable region of the kinetoplast minicircles (kDNA), amplified using the primers S35 and S36 [36]. PCR products were analysed by electrophoresis on 1.5 % agarose gels, stained with ethidium bromide and detected by UV light [34].

### Identification of *T. cruzi* DTUs and TcI SL-IR groups

To determine which TcI genotypes the *T. cruzi* strains obtained in this work belong to, a 350-bp fragment of the Splice leader intergenic region (SL-IR) was amplified using the primers TC2, TC1 and TCC and PCR conditions as reported in Souto *et al.* [37]. All amplicons obtained were sequenced in both directions (Macrogen Inc., Korea). Analysed samples included five isolates from *D. marsupialis* (including the sample from the scent gland secretion) and seven samples isolated from *T. maculata* captured in peridomiciliary environments.

For TcI genotype identification, representative sequences of each TcI SL-IR group [38–40] were retrieved from GenBank: TcIa SN8cl1, TcIb FChC and TcId PALC (access codes: EU127305, AM259469 and AM259473, respectively). Due to the reported presence of INDELs at the microsatellite region (positions 14–40) that generate multiple ambiguous alignments [41] that could lead to incorrect SL-IR group determination, we assigned samples to a specific SL-IR group according to the maximal nucleotide identity value derived from global pairwise alignments with the reference sequences.

## *T. cruzi* prevalence in children

### Blood sampling

#### Ethical approval

With previous written informed consent of one or both parents and following the requirements of the University of Antioquia Ethics committee (License 08-012-185), blood samples were obtained from 803 children under 15 years of age. The procedure was carried out in local hospitals and rural schools by trained personnel. Approximately 5 mL of whole blood was collected by venipuncture. Blood was centrifuged and serum was stored at −30 °C until processing.

### Serology analysis

Anti-*T. cruzi* IgG was detected by two serological tests: (i) all samples were subject to one initial screening by ELISA (enzyme-linked immunoabsorbent assay*)* based on crude parasites extracted from two *T. cruzi* isolates (I.RHO/CO/00/CAS-15.CAS; I.TRI/CO/03/MG-8.MAG). The optical density (OD) values of previously confirmed positive and negative controls were used to define the limits for seropositivity in this assay. OD values higher than 2 SD above the OD average for negative controls were considered ELISA-positive. (ii) All ELISA-positive samples were later evaluated by indirect inmunofluorescence (IFAT) with a titer of 1/40 used as the positive cut off. Samples were considered positive if both tests were reactive.

## Results

### Entomological survey, *T. cruzi* infection rate and blood-meal sources

**Fig. 1:**
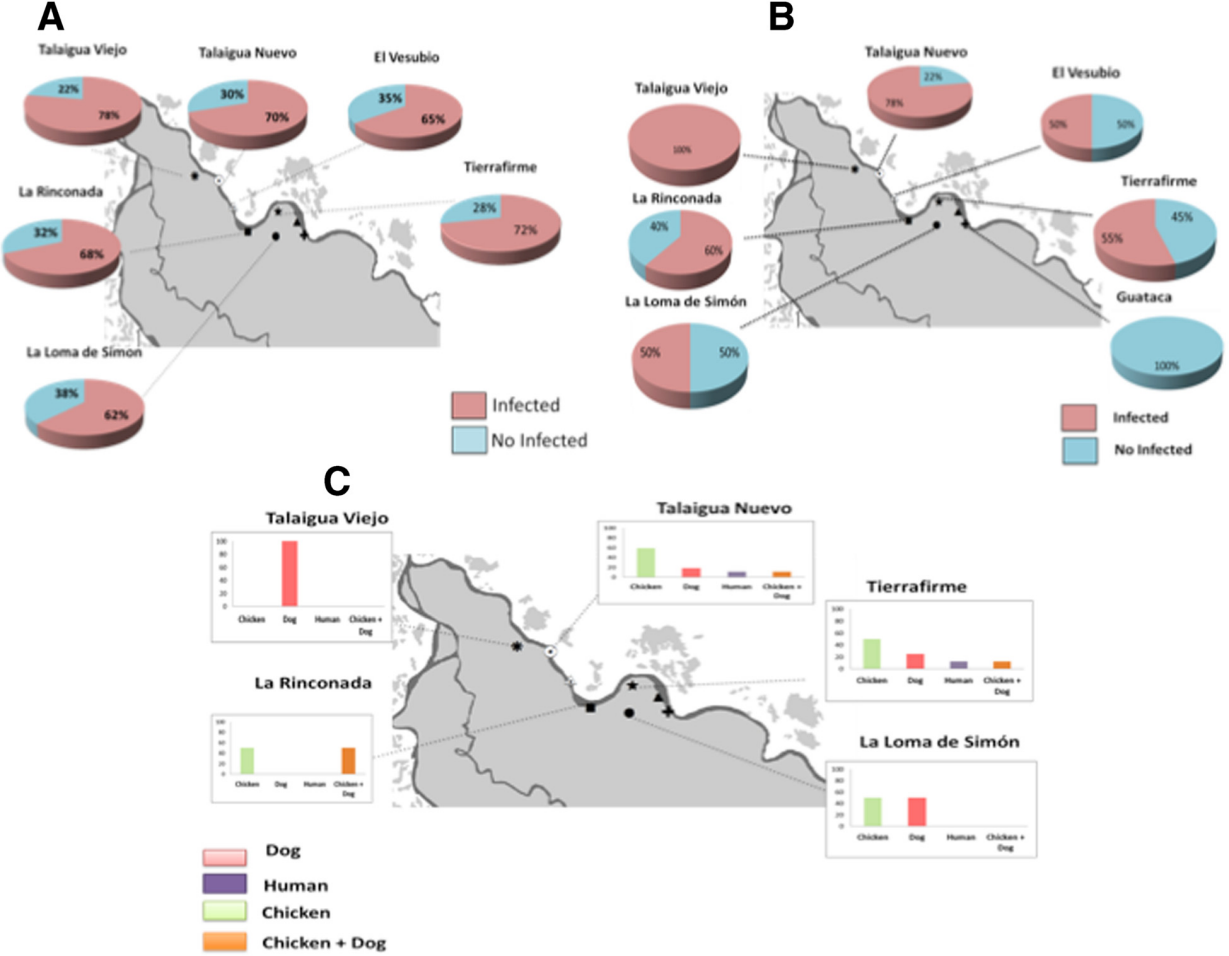
Study Area, including Talaigua Viejo, Talaigua Nuevo, El Vesubio, La Rinconada, Tierrafirme, La Loma de Simón and Guataca. **a** Prevalence *T. cruzi* infection of *T. maculata* by town. **b** Prevalence of infected dogs by town, and **(c)** frequency of blood meal source of *T. maculata* by town

A total of 124 insects were collected during this study, from 48 houses throughout all seven localities (Table 1), corresponding to an infestation prevalence of 10 %. The species captured were mostly (94 %) *T. maculata* (*n* = 117,) while the rest were *R. pallescens* (*n* = 6) and *E. cuspidatus* (*n* = 1). Active search in peridomiciliary environments from localities in TF and TN provided only 10 specimens (all instars of *T. maculata*), whereas most insects (mostly adults) were collected by the community, who brought them to receiving stations at all localities. All specimens were captured in peridomicile structures (mainly in pigeon and chicken coops) and no domestic infestation was detected. Of the 106 *T. maculata* specimens evaluated for *T. cruzi,* 76 were positive by PCR, which represents an average prevalence for all localities of 71.6 % (Fig. 1a). Additionally, 50 % (*n* = 3) of *R. pallescens* were positive, and the only *E. cuspidatus* captured specimen was not infected.

The HRM profiles of the cyt b sequences obtained from *T. maculata* guts or faeces indicated that chickens were the most common blood source (55 %), followed by dogs (20 %), and humans (10 %). Additionally, 15 % of *T. maculata* had mixed chicken and dog blood sources in several localities sampled (Fig. 1c). Interestingly, 17 of 23 (73.9 %), 4 of 9 (44.4 %) and 3 of 4 (75 %) triatomines fed on chicken, dog and human blood sources, respectively, were also infected.

### *T. cruzi* infection in dogs

The mean and standard deviation age of dogs was 3.65 ± 2.2 years (range from 6 months to 13 years). A total of 171 dogs (70.1 %) were seropositive for *T. cruzi* by both ELISA and IFAT tests, including six juvenile dogs (6–12 months old) (Table 1 and Fig. 1b). Co-infection with *Leishmania* spp was observed in 79 dogs, and 19 (7.8 %) dogs were seropositive for only *Leishmania* spp. Three dogs presented specific positive reactions against *L. infantum* as determined by the DPP test.

We found no significant difference in the *T. cruzi* infection rate according to the dogs’ sex, age (dogs ≤ 3 years old and older dogs) or the others variables evaluated in bivariate analysis used to identify risk factors. Overall, despite the high rate of *T. cruzi* infection in dogs, all haemocultures were negative and the presence of flagellates was not detected by direct observation of *R. prolixus* faeces from xenodiagnosis. However, *T. cruzi* DNA was successfully amplified by PCR in 45 % (9/20) of insects faeces from the xenodiagnosis bug pools.

### *T. cruzi* infection in synanthropic mammals

Thirteen *D. marsupialis* and ten *Rattus rattus* were captured in peridomestic areas.

Of the 23 synanthropic mammals tested using xenodiagnosis and haemoculture, eight *D. marsupialis* were positive for *T. cruzi* (Table 1)*.* Interestingly, a *D. marsupialis* captured in TN that tested negative in both xenodiagnosis and haemoculture assays, tested positive when a scent gland secretion was evaluated by satellite DNA 188 bp-fragment amplification (Data not shown).

### *T. cruzi* genotypes

All *T. cruzi* isolates from *D. marsupialis* and *T. maculata* were DTUI. A comparison of each sequence with reference sequences for each DTUI genotype showed that most isolates were TcIb, whereas the sample obtained from the opossum gland secretion was TcIa (Table 2). Strains isolated from *T. maculata* were mostly TcIb, and only two samples were assigned to a different SL-IR (7-TmTN6 to TcIa and TNA21 belonging to TcId) (Table 2).

**Table 2:** Description of TcI spliced-leader intergenic region (SL-IR) genotypes obtained from insect faeces

Host/vector	Locality	Code isolates	TcI SL-IR genotype (Identity percentage with reference sequences)
*D. marsupialis*	TF	XE6TF	Ib (93,6)
	TN	XE10TN	Ib (88)
		GlaD13TN	Ia (98,2)
	LS	XE11LS	Ib (88)
		XE12LS	Ib (87,8)
*T. maculata*	TN	3Tj1	Ib (97,8)
		5Tj11	Ib (99,6)
		6-TmTN	Ib (89)
		7-TmTN6	Ia (97,8)
		TNA21	Id (87.7)
		2MC09-92	Ib (84.4)
		1MC21-73	Ib (80.7)

### Seropositivity in children less than 15 years of age

A total of 803 children under 15 years old who lived in the 6 communities were tested, and only 2 were positive, which leads to an overall seroprevalence of *T. cruzi* of 0.25 % in this population. None of the children had a history of travel outside the study area or a blood transfusion, and their mothers were seronegative (Table 1).

## Discussion

We assessed the factors involved in *T. cruzi* transmission in peridomestic areas in the central zone of Margarita Island in the Colombian Caribbean region. We present evidence of the epidemiological importance of *T. maculata* and its participation in the *T. cruzi* transmission cycle within human households, as suggested by finding that infected insects feed on humans as well as evidence of recent transmission in children. Additionally, we described the role of dogs in peridomestic *T. cruzi* maintenance, and *D. marsupialis* as a bridge between peridomestic and sylvatic transmission cycles.

Traditionally, the apparent ornithophily preference of *T. maculata* has downplayed its role as a *T. cruzi* vector. However, recent studies have suggested its epidemiological relevance in the lowland Caribbean region of Colombia and the eastern Colombian plains [4, 5, 10, 42]. In a recent study [15], our group discussed the vectorial relevance of *T. maculata* based on a) the household-invading behaviour of infected adults, b) transmission in children less than fourteen years old, and c) an epidemiological association between the presence of this species in houses and human seropositivity [10, 15]. The present work provides new evidence supporting this argument. (i) We show the highest reported infection levels of household-invading *T. maculata* in Colombia, Venezuela and Brazil [5, 7, 9, 14, 16, 43]. (ii) The feeding patterns indicate that *T. maculata* is a generalist species with the ability to feed on different hosts, including humans. (iii) We found infected *T. maculata* that had fed on dogs and humans. This ecological behaviour has not been reported in other endemic regions such as Venezuela and Brazil, where peridomestic *T. maculata* is found with low or null *T. cruzi* infection, which is attributed to its ornithophilic preference [7, 14]. This evidence, coupled with the reportedly large number of blood meals taken during the nymphal cycle and defecation that occurs immediately after the blood meal [7], could explain the recently reported human infections in our study area, where this species has not yet been implicated as a *T. cruzi* vector [6, 10, 44]. (iv) We describe a well-established *T. cruzi* transmission cycle in the peridomiciles, including *T. maculata¸* dogs and synanthropic *D. marsupialis.* This is especially relevant for (re) interpreting the routes of canine infection because dogs may become infected by ingesting infected *T. maculata* [45] or ingesting infected small mammals [46]. (v) The analysis of SL-IR genotypes suggests a pattern of super-infection and/or co-infection as different genotypes are circulating in *T. maculata* populations*.* Peridomestic insects were found carrying sylvatic, peridomestic and domestic genotypes, such as TcIa, TcIb, and TcId, demonstrating the active participation of *T. maculata* in different *T. cruzi* transmission cycles. A limitation of this dataset is the impossibility of genotyping from the blood of positive dogs and humans due to limited amount of DNA shields in such samples. Subsequent studies in the area should direct the efforts in characterizing the genotypes of these hosts to be able to establish the ultimate relationship and intensity of transmission among triatomine vectors, dogs and humans in the area.

The importance of dogs in the transmission and maintenance of *T. cruzi* within households has been assessed in several studies that established that dogs may play different roles in different epidemiological scenarios, and should therefore be considered to be unique within a certain spatiotemporal scale. In Argentina, Gran Chaco dogs are highly infected by reported triatomine vector transmission, and therefore are considered the domestic reservoirs in this area [1]. A different scenario has been reported in Brazil, where transmissibility competence is rarely documented and dogs are considered to have little epidemiological importance or are sometimes reported to be a dead-end host [17, 19, 31]. The results of this work describe another role of domestic dogs in our study area, where dogs have low transmissibility competence but subpatent infectiousness to insects, and therefore could be considered to be a secondary reservoir of *T. cruzi* [47]. Simultaneous finding of positive PCR in insects and negative classic xenodiagnosis is likely related to a very rare bloodstream trypomastigote in mammals with the ability to maintain subpatent parasitemias [47, 48]. The high rate of dog-*T. maculata* contact corroborated with our blood meal source determination, suggesting that this relationship could explain the maintenance of *T. cruzi* in peridomestic habitats in this area. Moreover, the importance of a secondary reservoir could change according to other factors, such as health conditions, including immune suppression and concomitant parasitic infection [1].

The finding of PCR-positive xenodiagnosis insects gives further support to the fact that seropositive dogs were actually infected with *T. cruzi* and may eventually transmit it. In addition, molecular diagnosis allowed us to confirm *T. cruzi* infection in areas with co-circulation of related protozoan species that might cross-react in serologic tests. In the study area, the presence of *T. rangeli* and *Leishmania infantum* have been detected [49].

The role of domestic dogs as bioindicators of *T. cruzi* transmission risk to humans is an important tool of surveillance programmes in endemics areas. Serological tests show the exposure of dogs to *T. cruzi* infection, revealing the presence of parasites in areas close to humans and therefore the epidemiological risk of Chagas disease [1, 50]. In Colombia, rates of canine seroprevalence range between 1.42 % and 15 % in areas with domestic *R. prolixus*, such as the Boyaca and Tolima departments, suggested a moderate *T. cruzi* transmission cycle [23–25]. We report a canine seroprevalence of 71.3 %, suggesting widespread contact of dogs with a well-established peridomestic *T. cruzi* transmission cycle close to the human home. This finding is supported by the fact that 19 of 30 dogs under one year of age were seropositive, which indicates the occurrence of active transmission. Almost all (~90 %) of the infected dogs are used for house protection, and therefore restricted to peridomestic areas. In this sense, the low prevalence observed in children could be explained by the absence of triatomines and dogs inside the houses. However, a proportion of *T. maculata* fed with human blood, confirmed that a high risk of *T. cruzi* transmission could be related to the human infections reported here [1, 19, 31].

The presence of *Trypanosoma cruzi* blood-forms in peridomiciliary-collected *D. marsupialis* support their role as an amplifier reservoir of *T. cruzi* transmission in the peridomestic area. *D. marsupialis* is a recognized synanthropic animal, and as it approaches human dwellings, it may serve as a bridge between wild and domestic transmission cycles [51–53]. *T. cruzi* isolates from both opossums and vectors were mostly TcIb, a genotype previously found only in wild *R. pallescens* captured in an *A. butyracea* palm tree and in periodomestic *T. maculata* [15]*.* Another remarkable result is the positive infection detected in the scent gland secretion of one *D. marsupialis* individual captured in the TN locality. Infective *T. cruzi*-forms present in scent glands might confer a vectorial role for *D. marsupialis* as parasite transmission to humans and dogs can occur through food or skin contaminated with scent gland secretions [54, 55]*.* This sample was genotyped as TcIa, a genotype with a strong association with domestic cycles and reported to be the second most frequent genotype in oral outbreaks in Colombia [12]. However, *D. marsupialis* was not reported to be infected with *T. cruzi* in areas of Colombia where oral outbreaks occurred [12]. Our results suggest that the role of this synanthropic mammal could be underestimated in Colombian outbreaks because *D. marsupialis* is able to maintain *T. cruzi* infection in the glands without parasitemia [54].

Some authors have highlighted the competence of *D. marsupialis* as reservoirs in environments with a high degree of human activity [17, 56, 57]. Hence, this marsupial plays an important role in the amplification of the *T. cruzi* enzootic cycle or as a link between the enzootic and domestic cycles [17, 19, 31]. The presence of super-infection and/or co-infection in *T. maculata* could be a consequence of the high efficiency of *D. marsupialis* in introducing many *T. cruzi* genotypes in peridomestic areas [58, 59]. However, the blood-meal source analyses did not identify *D. marsupialis* as a blood source for *T. maculata,* which could be a result of our small sample size if *D. marsupialis* is not a frequent host, because the technique detects only the most recent blood meal [19]. Further studies using molecular epidemiology with multilocus microsatellites may be beneficial for understanding the role of this marsupial species in the local Chagas disease scenario.

## Conclusions

We show that despite the absence of *R. prolixus* and *T. dimidiata* as main vectors in the area, *T. cruzi* transmission is well established around homes, both in triatomine vectors and the canine population. Moreover, this complete *T. cruzi* eco-epidemiological profile shows that in an endemic area in Colombia, active transmission is associated with secondary vectors such as *T. maculata* and wild synantropic reservoirs that may also link sylvatic and domestic habitats. These results emphasize the need for continuous surveillance in areas without the main Chagas vectors*,* including the use of dogs-as-sentinels, active community participation in entomological surveillance programmes and ad hoc community-based interventions that organize and clear peridomestic areas to remove *D. marsupialis*.

## Additional file

Additional file 1:
**Geographic distribution of studied localities.**
